# Perspective: OLED Displays Singing with the Blues

**DOI:** 10.1002/adma.202519327

**Published:** 2025-12-26

**Authors:** Stephen R. Forrest, Claire Arneson, Haonan Zhao

**Affiliations:** ^1^ Department of Electrical Engineering and Computer Science University of Michigan Ann Arbor MI USA; ^2^ Department of Physics University of Michigan Ann Arbor MI USA; ^3^ Department of Materials Science and Engineering University of Michigan Ann Arbor MI USA

**Keywords:** exciton annihilation, Marcus theory, PHOLED, Purcell effect, TADF

## Abstract

While blue pixels consume approximately 50% of the energy of organic light‐emitting diode (OLED) display front planes, in the 25 years since their invention, 100% internal efficiency phosphorescent OLEDs (PHOLEDs) have not met the stability standards necessary for their adoption. In this perspective, we discuss the significant progress and challenges encountered during this long journey of exploration and discovery. Today, using a combination of solutions including robust molecular design, graded doping of the emission layer, increasing the optical density of states to decrease the triplet radiative lifetime, and employing light outcoupling schemes, deep blue PHOLEDs are now achieving lifetimes approaching those of their green analogs. This perspective elaborates on the challenges and opportunities confronting further development of triplet‐controlled emitters, including PHOLEDs using heavy‐metal phosphors and thermally activated delayed fluorescent OLEDs. We also address some persistent problems commonly found in the literature concerning the measurement of quantum efficiency and operational lifetime.

## Graphical abstract

While blue pixels consume approximately 50% of the energy of OLED display front planes, in the 25 years since their invention, 100% internal efficiency triplet‐controlled phosphorescent OLEDs have not met the stability standards necessary for their adoption. In this perspective, we discuss the progress and challenges encountered during this long journey of exploration and discovery. Using a combination of strategies, these devices are nearly ready to meet the most stringent demands of the highest quality mobile displays and televisions.

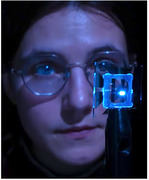

## 1 The Challenge of Stable, Deep Blue Phosphorescent Organic Emitters

Blue pixels consume approximately 50% of the energy used in an organic light‐emitting diode (OLED) display front plane. Therefore, it is no surprise that replacing the current blue fluorescent pixels with electrophosphorescent devices has dominated research in OLEDs since the first demonstration of a blue phosphorescent OLED (PHOLED) in 2001 [1]. Indeed, this may be the highest value and difficult to meet objective confronting the entire field of organic electronics. So what is holding the industry back from achieving this goal?

The problem was identified almost immediately following the invention of the blue PHOLED – it lacks the stability required for use in commercial display applications. Over the intervening 25 years, innumerable researchers in laboratories worldwide have attempted to extend blue PHOLED lifetimes to render them useful. Only now are we starting to see (blue) light at the end of the tunnel, with recent results showing significant promise for delivering highly efficient deep blue PHOLEDs whose lifetimes approach that of their green counterparts.

Approaches to extending the longevity of the emitters began as they had for both green and red PHOLEDs – by developing robust molecules with large bond dissociation energies (BDE). After all, blue photons have the highest energy (at ∼2.6 eV) within the visible spectrum, thus making fragile molecular bonds vulnerable to cleavage in the blue PHOLED emission layer (EML). But bond dissociation could not tell the full story. After all, fluorescent blue devices also containing fragile bonds were sufficiently durable to find use in both mobile and TV displays. This mystery was resolved seven years later by Giebink, et al. [2] who showed that the long triplet lifetime (∼several microseconds) results in a high density of triplets in PHOLED emission layers (EMLs). This, in turn, leads to a high probability for triplet–triplet and triplet–polaron annihilation (TTA and TPA, respectively, where a polaron is a free charge in an organic material). Annihilation reactions double the energy available for bond‐breaking. Such events can result in energies ∼6 eV, which is more than sufficient to break any molecular bond in the organic thin film whose BDEs typically lie in the range of 3.5–5 eV.

## 2 Early Solutions

While the work on finding more robust molecules continued apace, particularly with the introduction of tetradentate Pt‐complexes [3, 4, 5, 6, 7], the annihilation model suggested that purely chemistry‐based solutions would never suffice. After all, the bluer the photon, the higher its energy, and hence the more likely it is to break even the strongest bonds. And all the while, industry was demanding ever deeper blue emission to meet the requirements of the Rec. 2020 UHDTV color gamut. The prospect of using blue PHOLEDs in displays was looking very dim indeed. Yet the OLED community remained hopeful that better luck through clever chemistry alone would eventually solve the problem.

**FIGURE 1:**
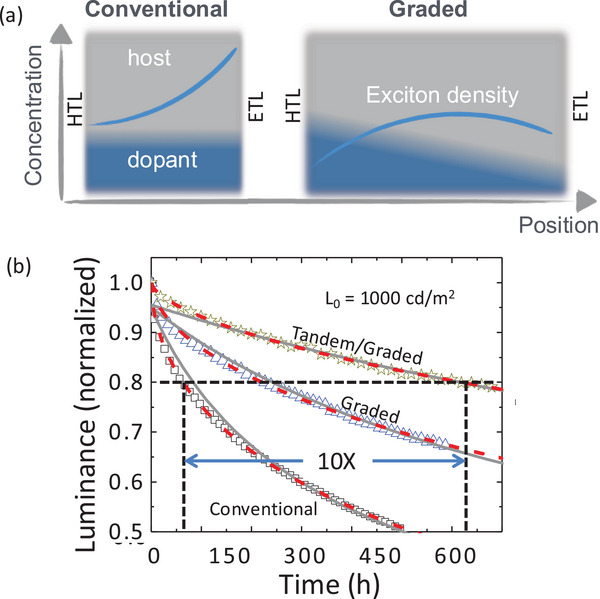
Graded doping of the emission layer (EML) extends device lifetime. (a) A conceptual picture of the exciton population (blue curve) across the EML for a conventional device with uniform grading of the dopant (blue shade) and host (grey shade), and a graded EML device. The pile‐up of excitons in the triplet population at the EML/electron transport layer (ETL) interface in the conventional device leads to enhance triplet–triplet and triplet polaron annihilation (TTA and TPA, respectively). The exciton density is lowered and spread across the EML between the ETL and the hole transport layer (HTL) in the graded device, reducing annihilation. (b) Luminance as a function of operation time of cyan‐emitting, Ir(dmp)_3_ phosphorescent OLEDs (PHOLEDs). There is a 10X increase in device lifetime for a tandem/graded PHOLED compared to a single‐element conventional OLED due to grading and stacking two PHOLEDs in the tandem structure. From [Ref. 8].

Validation of the annihilation picture finally came in 2014 when Y. Zhang and co‐workers [8] demonstrated that grading the Ir‐phosphor dopant concentration across the EML decreased the local density of triplets, thereby significantly decreasing the probability for TTA and TPA. This led to an increase in blue (in this case cyan) PHOLED lifetime by a factor of 3 compared to a conventional, uniformly doped PHOLED EML. Moreover, if two graded PHOLEDs were stacked in tandem, the triplet density needed to achieve the same brightness as a single stage PHOLED is cut in half, leading up to a tenfold increase in lifetime (see Figure 1). This demonstration was pivotal in that it began to move the research community toward searching for solutions that reached beyond solely chemistry‐based approaches.

An interesting question emerged with the introduction of the triplet annihilation model. While there is sufficient energy available to break almost any bond in the organic molecules in the OLED stack following annihilation of blue triplet excitons, this is also true of green excitons with energies of approximately 2.4 eV (and hence after annihilation, the excited state reaches nearly 5 eV). While the probability of the doubly excited state to couple completely to a molecular bond is low, green PHOLEDs should also be vulnerable to destruction at a regular rate following TTA and TPA events. But green PHOLEDs are known to last an exceptionally long time. Hence, a simple Arrhenius activation energy model could not explain the extraordinary fragility of blues compared to greens.

**FIGURE 2:**
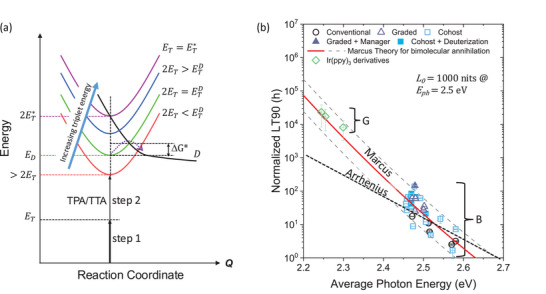
Degradation routes and the lifetime of PHOLEDs. (a) Energy environment leading to dissociation in PHOLEDs. Each parabola corresponds to the bound state energy surface vs. molecular configuration (the reaction coordinate, Q) for molecules whose emission energy ranges from red to deep blue (denoted by the corresponding color of each parabola). As the triplet energy, *E_T_*, increases, the transfer from the bound to dissociated state, D, occurs over a lower energy barrier of free energy, $\Delta G^{*}=E_{T}^{*}-E_{T}$ (dashed arrow). The dissociated state has no ground state, and hence the parabola is open‐ended (bold black line). Excitation into the precursor state is a two‐step process, the first of energy *E*_T_, and the second of approximately equal energy due to TTA or TPA. Here, $2E_{T}^{*}$ is the energy at which the energy barrier between the precursor and D vanishes. (b) The time for the luminance to decrease to 90% of its initial value, *L_0_*, is LT90 is plotted vs. photon energy, *E*_ph_, for a population of blue (B) and green (G) Ir‐based phosphor dopants. The LT90 values are normalized to the initial luminance excitance of each device. The solid line is a fit to the data using Marcus charge transfer theory, whereas the dashed line assumes an Arrhenius behavior for molecular degradation. Several different device architectures are included as noted in the legend. Adapted from [Ref. 11].

This apparent contradiction was recently resolved by returning to the basic principles of chemical reactions that resulted in a Nobel Prize in Chemistry awarded to Rudolph Marcus in 1992. He pointed out that chemical reactions, of which molecular dissociation is but one example, occur by a two‐step process shown in Figure 2a [9]. The first is excitation of the triplet at energy, *E_T_*, and the second is annihilation, yielding at least double that energy. The reactant and product states (i.e., the initial and dissociated states corresponding to the singly excited and dissociated emitting phosphor) must go through a doubly excited precursor state that shares the excitation. Whereas a simple Arrhenius process with activation energy equal to the triplet exciton energy, *E*_T_, occurs with a probability of $∼exp(E_{T}/kT)$, unimolecular non‐adiabatic Marcus theory [10] predicts a much steeper $∼exp(A{\left(E_{T}^{*}-E_{T}\right)}^{2}/kT)$ dependence. Here, $E_{T}^{*}$ is the energy needed to overcome the barrier to molecular dissociation even as *T* →0 (see Figure 2a), and *A* depends on molecular structural details. Also, *k* is Boltzmann's constant and *T* is the temperature. The quadratic dependence on *E*_T_ clears up the mystery of the unexpectedly high probability for degradation of blue phosphor molecules through annihilation events. And the deeper the blue demanded by the display, the smaller the activation energy, $\Delta G^{*}=E_{T}^{*}-E_{T}$ for dissociation (cf. Figure 2a), leading to a lower possibility for achieving an acceptable operational lifetime.

The different predictions of Arrhenius and Marcus processes are shown in Figure 2b, where LT90 (i.e., the time it takes for a PHOLED to lose 10% of its initial luminance of approximately 1000 nits) is plotted vs. triplet energy for a population of green and blue Ir‐based phosphors [11]. It is apparent that Marcus theory, which inherits its quadratic energy dependence from the parabolic energy surface of the precursor and product states, is consistent with the rapid photon energy dependence of both green and blue phosphors, whereas the simple Arrhenius picture does not provide an adequate fit to the data over such a large energy range.

Thus, we can justifiably ask whether the quest for long‐lived blue triplet‐controlled devices is futile.

## 3 Breaking the Lifetime Bottleneck with Polaritons

With this theoretical background and a wealth of practical experience, it becomes clear how to fix this exasperating blue problem. Yes, we need more robust materials – a problem that requires yet more attention from the chemistry community. But we also must reduce the density of triplets within the device stack. This latter solution creates an unfortunate dichotomy. While fewer triplets result in reduced annihilation and hence the probability for molecular destruction, fewer triplets might also result in lower intensity. Zhang's experiments with graded EMLs are a step in the right direction, but a mere 10× increase in lifetime is not nearly adequate. To gain parity with green PHOLED lifetime, Figure 2 shows that at least a 1000× increase is required. So, how is the triplet density decreased without compromising other performance characteristics of the devices? These are a few of the methods that can be employed:

1) Decrease the triplet lifetime via stronger spin‐orbit coupling. This has been attempted for heavy‐metal phosphors based on Ir or Pt, but it does not seem possible to reach radiative lifetimes less than 1 µs, which is not nearly enough [12]. Furthermore, metal‐containing thermally activated delayed fluorescent (TADF) emitters have achieved triplet lifetimes as short as 250 ns [13, 14], although this too is not short enough and the molecules (based primarily on Au or Ag) have intrinsically weak bonds.
2) Eliminating the doubly excited annihilation products using a third “triplet manager” molecule in the EML is a possibility, but again it has not proven sufficient to reduce the hot excited state population to levels that can lead to acceptably stable blues [15].
3) Increasing the optical density of states (ODOS) in the OLED optical cavity. This increases the pathways for radiative relaxation of triplets such that their density decreases without a penalty to other performance parameters such as quantum and power efficiency.

The mechanism for decreasing triplet lifetime without sacrificing efficiency is derived using one of the most basic principles of quantum mechanics: Fermi's golden rule (FGR). This provides a link between the exciton radiative decay rate (*k_r_*) and the ODOS, viz.:

(1) $$k_{r}=\frac{2\pi }{\hbar }M_{\mathrm{if}}^{2}\rho \left(E_{\mathrm{ph}}\right)$$

where *M*_if_ is the transition matrix element coupling the initial (*i*) and final (*f*) states of the exciton, and *ρ*(*E*_ph_) is the ODOS of the initial and final states of a photon at energy, *E*_ph_. Also, ℏ is the reduced Planck's constant. An optical cavity can increase *ρ*(*E*_ph_), and therefore increase *k*_r_ which in turn decreases the triplet density without changing the efficiency of the emission. The ‘strength’ of the cavity is expressed by its Purcell factor (PF). That is, for a molecule with a natural emission rate of *k_r0_*, the rate in the cavity is given by:

(2) $$k_{r}=PF\;k_{r0}$$

In this context, OLED optical cavity engineering has shown the greatest promise, especially when combined with dopant and host grading, and robust emitting molecules.

Since the triplet density is a function of the current, the operational lifetime of the device also must take that factor into account. That is, the lifetime decreases with increasing current, and hence the brightness necessary for a particular application. The lifetime, LT*x*, is defined as the time it takes for the brightness at a constant current density, *J*, to decrease to *x*% of its initial luminance, *L_0_* (i.e. *L*_0_∝*J*). Combining the dependence of current, cavity effects, and triplet energy as discussed above, we obtain an approximate expression:

(3) $$LTx\propto PF^{m}J^{-n}\exp\left[A{\left(E_{T}^{*}-E_{T}\right)}^{2}/kT\right]$$

here, 1 < *m* < 3 and 1 < *n* < 2 are empirical exponents that depend on the details of the annihilation process. For example, if annihilation is only via TPA, *m* →1, and via TTA, *m* can be as high as 2.5 – 3 [11].

The most effective use of the Purcell effect is to “amplify” it by the excitation of polaritons that couple the singlet in the transport layer with the surface plasmon in the adjacent metal contact. A polariton is a quantum state that comprises a cavity photon coupled to an exciton. Two coupled quantum mechanical oscillators (i.e., the cavity photon and the exciton) result in two normal modes of oscillation separated by energy, ℏΩ, where Ω is the Rabi frequency. The two modes individually occupy the lower (LP) and upper polariton (UP) states. Polaritons in OLED optical cavities have been exploited to achieve narrow spectral emission [16, 17, 18, 19].

Relevant to this discussion, the largest enhancement that has brought blue PHOLED lifetimes close to that of green phosphorescent devices uses the so‐called plasmon‐exciton‐polariton (or PEP) effect.

A PEP is formed when the high oscillator strength absorption transition from a molecule in the transport layer is coupled to the surface plasmon polariton (SPP) mode excited in the nearby electrode metal (preferably Ag due to the strength of its plasmon in the blue‐green spectral region). Here, an SPP is a short‐range, collective excitation of charge at a metal surface induced by an incident electromagnetic wave [20, 21]. While SPPs are generally lossy due to the finite resistance of the metal, at the same time, they increase the ODOS. Thus, transfer of energy from the EML to the SPP can reduce the density of excitons, and hence the probability for losses due to annihilation. Note that since coupling to plasmons is via a very short‐range interaction, ordinarily the emitting molecule must be placed very close to the electrode (i.e., within a few nanometers)[21, 22, 23]. This juxtaposition places unreasonable constraints on OLED design since the charge transport layer positioned between the electrode and EML must be sufficiently thick to be continuous and to prevent the excitons in the EML from quenching at the electrode. Furthermore, the EML itself is typically ∼50 nm thick, implying that many emitting molecules may be 80 – 100 nm distant from the electrode, making their coupling to SPP modes negligible [20].

**FIGURE 3:**
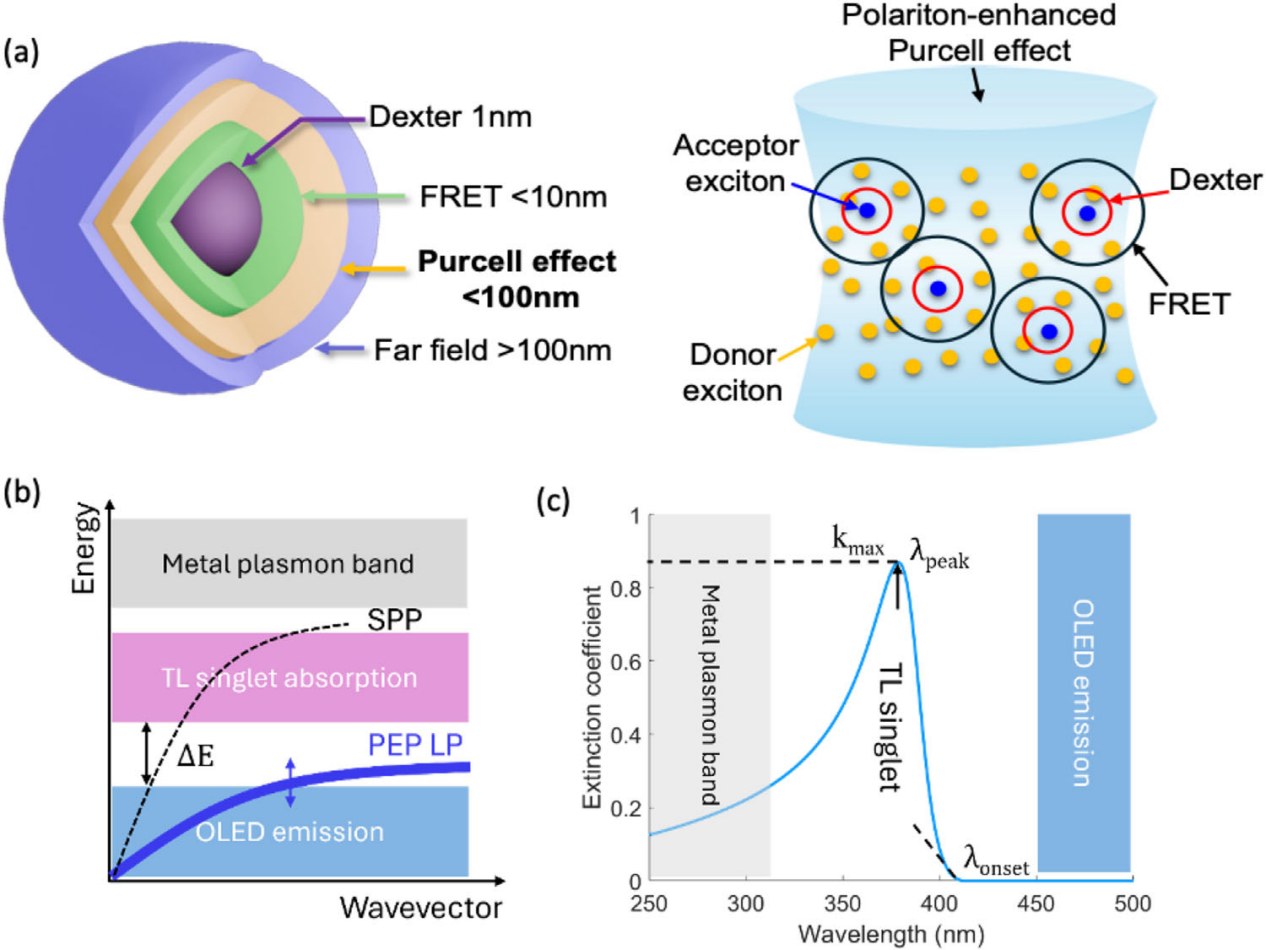
The plasmon‐exciton‐polariton (PEP) OLED concept. (a) *left*: Several ranges of exciton energy transfer. Dexter transfer (1 nm) requires contact between neighboring molecules, Förster resonant energy transfer (FRET) is an electrostatic mechanism with a range of ∼10 nm, and far field transfer (>100 nm) occurs when light from an emitting molecule is absorbed by another following the Beer‐Lambert law. Energy transfer induced by PEP formation occurs over a distance intermediate between FRET and far field transfer. *Right*: Visualization of the relative ranges of energy transfer within the PHOLED EML. From [Ref. 27]. (b) Dispersion relationships of the surface plasmon polariton (SPP) and lower polariton (LP) branch of the PEP, compared to the PHOLED triplet emission in the blue, and the transport layer (TL) singlet absorption in the near UV. Also shown is the higher energy plasmon band in the metal electrode. (c) Energetics of the PEP system. Here, *k*_max_ is the maximum extinction coefficient of the TL singlet at a peak wavelength of *λ*_peak_. To avoid loss, the onset of the TL singlet absorption, *λ*_onset_, should not overlap the blue triplet spectrum.

The ranges of energy transfer via different mechanisms are illustrated in Figure 3a. Typically, exciton coupling to SPP modes occurs by near field dipole interactions. However, if polaritons couple to the triplet in the EML, the energy can be transferred through strong, polaritonic coupling between the SPP and the singlet in the transport layer. This “double energy transfer” inherent to the so‐called “PEP effect” allows for efficient excitation across a typically 20 – 40 nm thick transport layer and the EML without compromising other OLED performance parameters.

Implementation of the PEP effect is a simple matter of finding a transport layer material whose strong singlet absorption lies between the triplet exciton emission and the SPP mode in the contact, as shown in Figure 3b,c. The long wavelength edge of the singlet absorption spectrum bends the PEP LP branch toward the triplet energy, enabling efficient transfer via weak coupling between the PEP and the triplet. The short wavelength tail of the singlet overlaps the SPP mode in the electrode, ensuring strong coupling to plasmons in this two‐step process. Indeed, the absorption at short wavelengths can also couple to the very short wavelength emission of some blue emitters, thus reducing or eliminating the density of the most destructive highest energy excited states.

The PEP effect extends device lifetime by significantly increasing the ODOS. It is further strengthened by a weak optical cavity in the OLED stack. All OLEDs form cavities with PF > 1, albeit the increase is small. By employing semi‐reflective anode and cathode contacts, PF > 3 can be achieved. This also increases OLED quantum efficiency along with spectral narrowing that results in more saturated deep blue emission compared with the natural emission spectrum of the chromophore. All of these advantages combine to result in increases in blue PHOLED lifetime beyond that achieved by any other method (including phosphor molecular engineering, use of EML co‐hosts using different molecules to conduct electrons and holes in the EML [24], and EML dopant grading) attempted to date.

**FIGURE 4:**
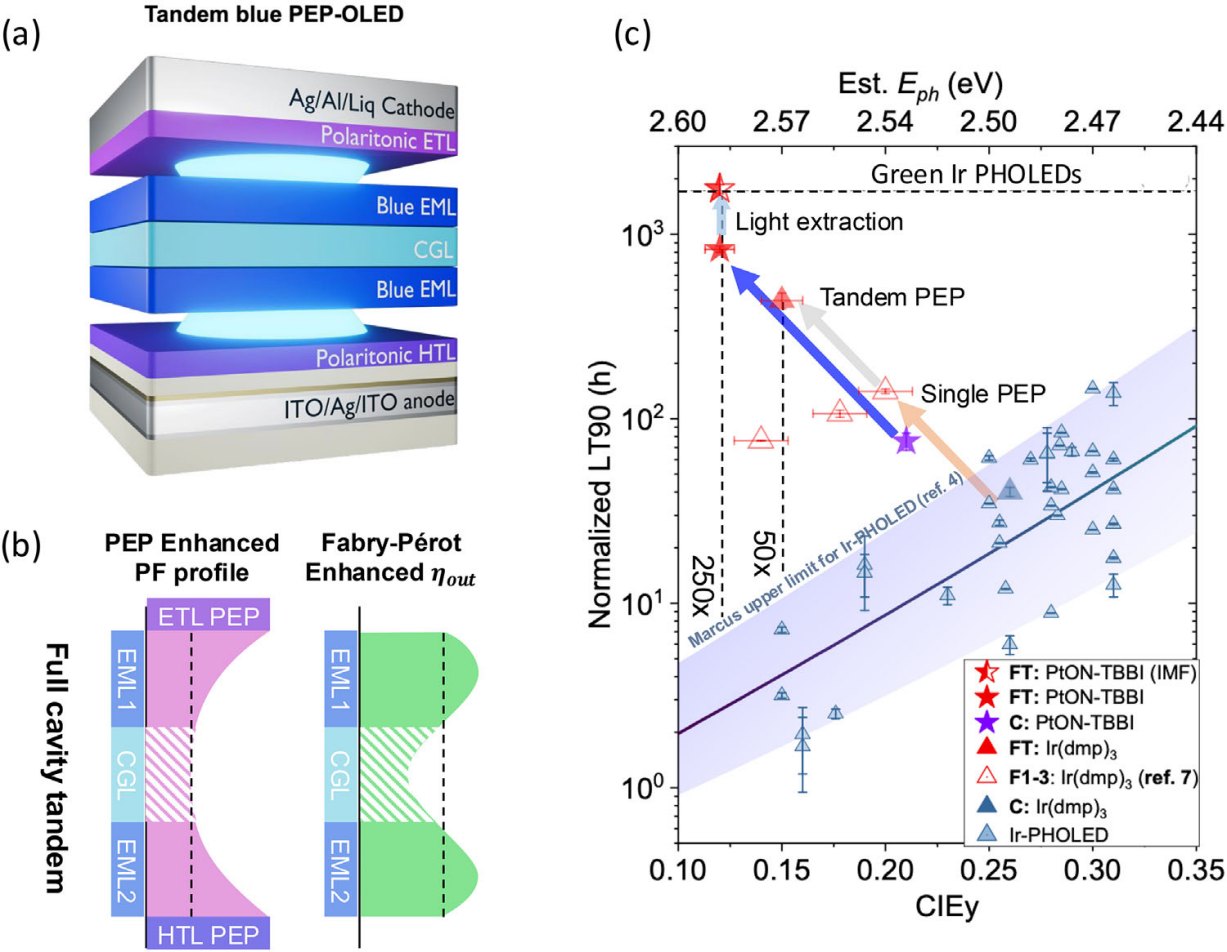
Very long lived tandem blue PHOLEDs using the double‐sided PEP effect. (a) A tandem blue PHOLED using a HTL near the anode and an ETL near the cathode that both support the formation of a PEP according to the criteria in Figure 3. Here, CGL is the transparent charge generation layer sandwiched between the two PHOLEDs in the stack, and whose thickness is adjusted to maximize (b) the Purcell factor (PF) across both EMLs, as well as their outcoupling efficiencies, *η*_out_. (c) LT90 of the population of blue devices in Figure 2 vs. their CIE*y* coordinate (bottom axis), and corresponding photon energy along the top axis. LT90 is normalized to an initial excitance of *M_0_* = 1000 nits at CIE*y* = 0.30. Also shown are enhancements using single sided Ir(dmp)_3_ PEP‐enhanced PHOLEDs (open triangles) and an analogous tandem device (closed red triangle). Also shown are data for a conventional PtON‐TBBi PHOLED (purple star), as well as the PEP‐enhanced tandem with and without light outcoupling from the substrate. The dashed horizontal line is the lower limit for green Ir‐based PHOLEDs, also shown in Figure 2. The solid line is from Marcus theory showing the 95% confidence limits (shaded region). The enhancement in LT90 for the PtON‐TBBi and Ir(dmp)_3_ PEP‐enhanced tandems compared to conventional, single element devices taken from the upper confidence limit, are 250X and 50X, respectively. From [Ref. 26].

Figure 4 illustrates the design principles and benefits that result from PEPs employed in a tandem blue PHOLED. In this two‐EML device, both the hole and electron transport layers adjacent to the anode and cathode, respectively, have the properties in Figure 3b that promote polariton generation. Furthermore, the anode, which typically comprises indium tin oxide (ITO), consists of a thin layer of Ag sandwiched between two ITO layers due to the strong SPP formation properties of this metal. Otherwise, the EMLs assume a conventional PHOLED design employing the cyan‐emitting Ir(dmp)_3_ or the more robust and slightly bluer PtON‐TBBi. Sandwiched in the center of the tandem is the transparent charge generation layer (CGL) whose thickness is adjusted to maximize both the PF and outcoupling efficiency *(η*_out_). Indeed, Figure 4b shows that having a PEP transport layer on both sides of the device leads to a high, and nearly constant PF across both EMLs while simultaneously optimizing *η*_out_. Apparently, there is considerable device design flexibility afforded by varying the CGL thickness.

The performance improvement of the tandem with an enhanced Purcell effect is shown in Figure 4c, which plots LT90 vs. 1931 Commission Internationale d'Eclairage (CIE) y‐coordinate (bottom axis) and the photon energy, *E*_ph_ (top axis). The triangles are values of LT90 obtained for various Ir‐complexes, and the stars are for PtON‐TBBi. The solid line shows the result from Marcus theory taken from Figure 2b.

In compiling these data, we make two important comments. In spite of there being standardized methods of measuring OLED quantum efficiency [25], many laboratories neglect to follow accurate measurement approaches. For example, reports often measure the luminance using a luminance meter with a very narrow angle of view. Then the assumption is made that the emission spectrum of the OLED is a Lambertian (which it never is) and the luminance meter captures all the spectral content of the emission (which it doesn't). This method is, therefore, unreliable, at best. Worse still, some efficiencies are quoted at very low intensities (and sometimes below the noise floor of the detector!), which are irrelevant to characterizing devices for practical applications. The second challenge is in comparing the LT90 from measurements of devices taken at different initial luminance, current, and test period. For example, reported measurements often assume different LT*x*, with *x* ranging from 50 to 95. To plot these data on the same axis, one needs to assign a lifetime exponent, *n* (Equation 3), which may not be precisely known for a given material and diode structure. Also, LT*x* is measured from an initial luminance, *L_0_*, which depends on a precise knowledge of the emission spectrum. Hence, in Figure 4c, we normalize the data to the OLED excitance, *M = EQE • J*, which quantifies the total number of photons emitted.

When we take these factors into consideration, Figure 4c shows the improvement in device lifetime compared to conventional single junction devices based on Ir(dmp)_3_ and the longer lived and slightly deeper blue PtON‐TBBi [26]. The improvements are remarkable, yielding conservative estimates of 50X and 250X lifetime improvement for the Ir and Pt complexes, respectively, when normalized to the same CIEy and employed with light outcoupling structures. Indeed, the Pt complex utilized in a tandem with substrate mode outcoupling yields a deep blue CIEy = 0.11, and LT90 = 1800 h, equal to the lower lifetime limit of current green PHOLEDs used in commercial displays.

Interestingly, the PEP effect has also shown significant improvements in blue phosphor sensitized fluorescent (PSF) [27] devices. In fact, the Purcell and PEP effects can improve the intrinsic device lifetimes based on any triplet‐controlled emission process (e.g., PHOLEDs, PSF, TADF, and TADF‐sensitized fluorescent – TSF — OLEDs) since it accelerates the excited state emission rate. This, in turn, reduces the exciton density and annihilation rate regardless of the origin of the radiative state.

## 4 Where We Go From Here

At the beginning of this Perspective, it was stated that achieving a blue phosphorescent OLED is possibly the most daunting yet important challenge confronting the field of organic electronics. After more than two decades of research, a solution to the problem remains elusive, but nevertheless is well within our sights. Indeed, there were many years during this long journey where solving the problem seemed hopeless, although it has continually approached a solution by taking many incremental steps, from molecular discovery to optical cavity engineering. Arguably, the biggest stride made was to employ an entirely quantum mechanical phenomenon, the polariton, as an effective route to radically decreasing the triplet density within the PHOLED EML, and thus limiting high‐energy annihilation. But none of the steps taken could have been accomplished without first having a fundamental understanding of why triplet annihilation damages molecules in the first place. The most worrisome aspect of the energy‐based annihilation model is that the deeper the blue emission, the more likely it is for the molecules to be destroyed. In this context, displays require very deep blue phosphors (CIE*y* = 0.046 is required to meet Rec. 2020 standards), and hence is the hardest application to satisfy. Yet only a blue‐green emitter is required for lighting, which apparently is now ready for deployment for interior illumination.

One lesson taken from the journey to a stable blue, triplet‐controlled emitter, is that several solutions are multiplicative, and, therefore, mutually supporting. A compilation of significant advances made in improving blue, triplet‐controlled OLED lifetimes is provided in Table 1. These solutions include developing robust, mid‐ to deep blue‐emitting molecules, reducing the triplet density via developing molecules with large radiative rates, grading the EML hosts and dopants to spatially disperse the excitons, using optical cavities to increase the radiative rate based on the Purcell effect, and using tandem PHOLEDs and light outcoupling methods to increase the EQE. Employing several of these solutions in combination, deep blue (though not yet saturated enough for high‐quality displays) PHOLED operational lifetimes are now approaching that of green devices used in OLED TVs and mobile appliances. Indeed, the list of solutions is quite long and is by no means fully explored. There is even more progress to be made, reassuring the community that the path to acceptable blue stability has not yet come to an end.

**TABLE 1:** Progress in extending the lifetime of triplet‐controlled OLEDs.

Year	Innovation	Emitter	CIEy	*J* (mA cm^−2^)	EQE (%)	LT90 (h)	Normal. LT90 (h)^a^	Lifetime Enhance^b^	Refs.
2001	Blue PHOLED	FIrPic	0.29	5	6	—	—	—	[1]
2008	Blue PHOLED lifetime	Ir(dmp)_3_	—	7	9	35	48	0.9	[2]
2014	Graded doping	Ir(dmp)_3_	0.31	6	10	60	69	2.2	[8]
2016	Tetradentate Pt complex	PtNON	0.21	20	8	16	116	6.8	[4]
2017	Hot excited state manager	Ir(dmp)_3_	0.30	5	10	141	145	4.8	[15]
2018	Phosphor‐sensitized fluor. (PSF)	DtPAPy + Ir phosphor^c^	0.27	25	10	10	193	7.4	[28]
2020	Purcell Enhanced PHOLED^d^	Ir(ppy)_3_	0.61	80	7	85^e^	6577	3.0	[22]
2021	Deuteration	Ir2D	0.28	2	22	75	59	2.1	[6]
2024	PEP‐enhanced single junction	Ir(dmp)_3_	0.20	7	10	110	216	13.6	[31]
2025	PEP‐enhanced PSF	ν‐DABNA +PtON‐TBBi	0.09	10	17	56	508	103	[27]
2025	Purcell‐enhanced metallorganic TADF^f^	(MAC)Cu(ph_2_Cz)	0.55	5	10	64	61	1.3	[29]
2025	PEP‐enhanced Tandem	PtON‐TBBi	0.12	3	35	215	830	112	[26]

^a^ LT_90_ extracted from the literature is normalized to an initial photon excitance *M_p,0_* = 50 µmol s^−1^m^−2^ where *M*_p,test_ = EQE×*J*: $LT90=LT90_{test}{\left(\frac{M_{p,\mathrm{test}}}{M_{p,0}}\right)}^{n}$. The acceleration factor *n* = 1.8 is based on standard values in the literature [7, 8, 31, 32]. The normalized excitance *M*_p,0_ corresponds to an initial luminance *L_0_ =* 1000 cd m^−2^ at CIEy = 0.3, and *L_0_ =* 500 cd m^−2^ at CIEy = 0.12 [see [Ref. 9, 10]];

^b^ The lifetime enhancement is calculated relative to the upper bound of the Marcus theory fit for Ir‐based OLEDs in Figure 4c;

^c^ Tris[1,2‐phenylene(3‐phenyl‐1H‐imidazo[4,5‐b]pyrazin‐1‐yl‐2(3H)‐ylidene)]iridium;

^d^ Green emitter;

^e^ Value extrapolated from LT95;

^f^ Green emitter. The Purcell‐enhanced TADF lifetime enhancement is calculated relative to a control (no Purcell enhancement) TADF utilizing the same emitter.

Finally, it is noteworthy that beyond heavy metal phosphors, many alternative approaches to light emission have been explored for their potential for higher stability. These include PSF [27, 28], TSF, TADF OLEDs [29], and most recently in combination fluorescent/phosphorescent tandems [30]. The longest lived blue OLEDs thus far demonstrated are the PEP‐assisted tandem PHOLED and single element deep blue PSF OLED. Note that if placed in a tandem structure, which is now the standard in commercial displays, the PSF OLED lifetime extrapolates to a 2X increase in enhancement compared to the Marcus theory projection to 206X over the single element device (see Table 1). This suggests that LT90 = 1015h at CIEy < 0.09 without outcoupling enhancements such as microlens arrays, which is within easy reach. Nevertheless, to date, PHOLEDs remain the emitter of choice due to their high efficiency, saturated color emission, and greatest stability among all these alternatives.

## Conflicts of Interest

The authors declare the following competing financial interest(s): S.R.F. has an equity interest in Universal Display Corp. This apparent conflict is under management by the University of Michigan Office of Research. Also, University of Michigan has a license agreement with UDC.
